# Unravelling the structure of monocyclic carbon ring cations using vibrational spectroscopy

**DOI:** 10.1039/d6cp03280j

**Published:** 2026-10-05

**Authors:** Thomas E. Douglas-Walker, Amit Debnath, Olivia O'Neill, Ewen K. Campbell

**Affiliations:** a School of Chemistry, The University of Edinburgh Joseph Black Building, David Brewster Road, King's Buildings Edinburgh EH9 3FJ Scotland UK e.k.campbell@ed.ac.uk

## Abstract

Laser ablation of graphite produces a diverse range of molecular structures including chains, rings and fullerenes. Whilst several of these species have been spectroscopically characterised and detected in the interstellar medium, until recently, the spectra of many monocyclic rings were unknown. In the infrared (IR), action spectra of C_4*n*_^+^ rings (*n* = 5–11) have been shown to exhibit giant IR resonances, which arise from a Kekulé-type vibrational mode. However, the structure and spectroscopic properties of smaller C_4*n*_^+^ rings are unexplored. In this study, the IR spectra of C_4*n*_^+^ rings (*n* = 2–7) have been recorded using He-tagging spectroscopy. The most intense IR mode of the larger monocyclic rings (*n* = 4–7) show systematic size-dependent trends, where a linear redshift and increase in intensity is experimentally observed as the ring size increases. A breakdown in these trends is observed for smaller clusters – whilst C_12_^+^ deviates slightly, C_8_^+^ shows more substantial differences, indicating a departure from their common structure. Density functional theory calculations suggest these observations are largely due to changes to the bond length alternation of the polyynic species, however, the exact structures are challenging to model using these methods. The results provide new insights into the geometric structure of carbon cluster cations along with the requisite spectroscopic data to search for these species in astrophysical environments.

## Abstract 2

Gas phase spectroscopy of monocyclic carbon ring cations (C_4n_^+^, *n* = 2–7) reveal intense infrared transitions due to Kekulé-type distortions. A breakdown in linear trends is observed for C_12_^+^ and smaller rings, indicating a change of common structure.

## Graphical abstract

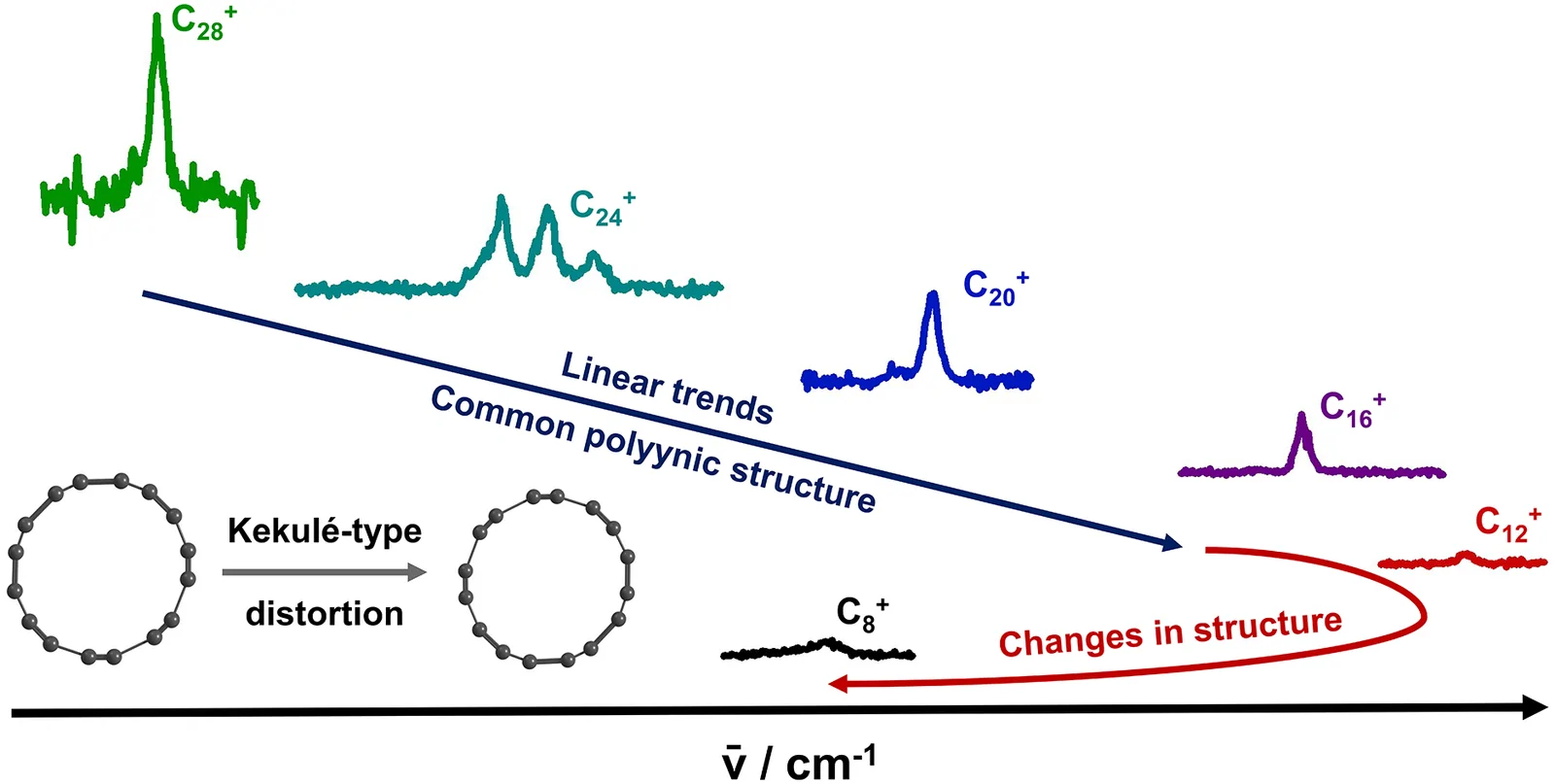

## 1 Introduction

In addition to traditional carbon allotropes, graphite and diamond, carbon can exist in a rich assortment of nanostructures. Many of these motifs can be generated by laser ablation sources containing pulsed nozzles^1,2^ which were initially used for the production of gas phase metal clusters.^3,4^ Laser ablation of graphite targets has been crucial for the generation and characterisation of new carbon allotropes, including fullerenes such as C_60_,^5,6^ whose structure was confirmed following its macroscopic synthesis.^7^ Efforts to resolve the configuration of other, smaller carbon clusters were strongly aided by ion mobility studies.^8–10^ These experiments indicated that, for cations, linear carbon chains exist up to C_10_^+^, planar ring structures appear from C_7_^+^ and exist beyond C_40_^+^, bicyclic rings are observed from C_21_^+^ to at least C_40_^+^ and the smallest fullerenes are observed at C_30_^+^.^11^ These observations are in agreement with reactivity studies,^12–14^ free energy arguments,^15^ and calculations,^16,17^ which suggested the presence of multiple isomers with abundances highly-dependent on the number of carbon atoms.

Despite representing the most stable form of carbon for clusters containing ∼10–30 atoms, the properties of monocyclic rings are still not fully understood.^18^ Importantly, the exact geometric structure of these cyclocarbons remains to be determined. They have been proposed to adopt one of four structures: the ring can either exhibit cumulenic or polyynic bonding, with equal or alternating bond angles (see, for example, ref. 19 and 20 for a comprehensive review on this topic). A diagram of these proposed structures is illustrated for C_12_ in Fig. 1. The distortion from a cumulenic, fully symmetric arrangement (structure A), to one that exhibits bond angle alternation (structure B) or bond length alternation (structure C), arises from a Jahn–Teller effect^21^ or a Peierls-type distortion,^22^ respectively.^23,24^ A combination of these effects is also proposed to occur, leading to structure D.

**Fig. 1:**
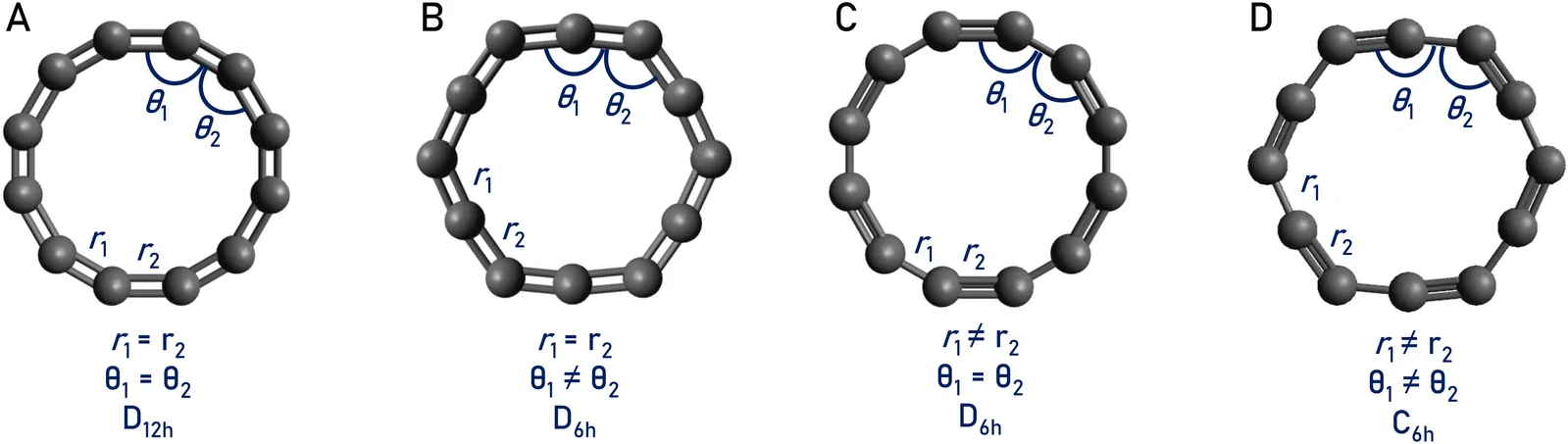
Diagram of four suggested structures of monocyclic carbon rings using C_12_ as an example. Structure A is the fully symmetric structure, structure B exhibits a bond angle alternation due to a second order Jahn–Teller effect, structure C shows bond length alternation due to a Peierls-type distortion and structure D shows the lowest symmetry, polyynic form with bond angle alternation.

In computational chemistry, obtaining accurate geometries of monocyclic rings is a widely-discussed challenge.^17,25–32^ The choice of computational method has a significant impact on the predicted structure, with pure density functional theory (DFT) calculations favouring a cumulenic structure, whereas Hartree–Fock (HF) and coupled cluster methods tend to yield polyynic bonding. Recent hybrid DFT methods including HF exchange have also predicted polyynic structures, with a greater HF exchange weight favouring this alternating bond length arrangement.^33^ Following the isolation of C_18_ on a surface, its polyynic form was observed by atomic force microscopy (AFM) imaging.^34^ However, this study was not able to resolve if any bond angle alternation (BAA) was present. Many carbon rings have now been imaged using similar methods, showing a clear bond length alternation (BLA) for many systems.^35–42^ Interestingly, some smaller C_4*n*+2_ rings appear to form cumulenic structures on a surface, namely C_6_, C_10_ and C_14_.^43^

Cationic carbon rings are of additional interest since ionisation will cause changes to their electronic and geometric structure. Calculations on open shell systems have additional challenges; for example, multireference effects mean geometry optimisations using some functionals give severe spin contamination. This includes popular methods such as CAM-B3LYP, *ω*B97XD and BHandHLYP.^18,44^ The challenges and discrepancies of calculating molecular parameters of carbon rings, in particular cationic rings, mean experimental data is strongly warranted.

Spectroscopy is a powerful probe to investigate the geometric and electronic structures of these clusters. Until relatively recently, most studies had been carried out using matrix isolation. In these experiments, the different reactivities of linear and cyclic isomers were used to aid the assignment of absorption features.^45^ More recent studies, combining mass spectrometry with cryogenic ion trap storage devices, have facilitated the measurement of radical cations using messenger-tagging spectroscopy.^46,47^ It is worth mentioning that significantly more studies have been published on other carbon clusters including linear chains^45,48–59^ and fullerenes,^60–69^ however, these will not be discussed in detail here and readers are instead referred to the cited works or relevant review articles.^70–73^

Electronic spectra have been obtained for various monocyclic rings, such as C_10_, C_12_ and C_6_^+^–C_9_^+^, using matrix isolation.^45,53,74^ Note that a later study suggested that C_10_ and C_12_ may have been misassigned.^59^ Electronic absorptions of C_14_, C_18_ and C_22_ have also been reported in the gas phase.^23,75^ The authors observed a distinction between the spectrum of C_14_, and that of C_18_ and C_22_,^23^ which could be due to a change from cumulenic to polyynic bonding arrangements, as was observed on a surface.^43^ Additional studies in the gas phase have re-investigated C_6_^+^,^46,51,76^ however concensus does not seem to have been reached concerning the structural assignment. A recent spectrum of C_4_^+^ has also been tentatively attributed to a cyclic isomer.^51^ The gas phase electronic spectra of larger C_4*n*_^+^ and C_4*n*+2_^+^ carbon rings, recorded by helium-tagging,^25^ nitrogen-tagging^77^ and two-colour fragmentation^78^ showed a linear red-shift of the origin band as the ring size increased. The origin band for the C_4*n*_^+^ species were consistently broader than those of the C_4*n*+2_^+^ structures, presumably due to more rapid non-radiative decay from the excited state.^17,77^ Interestingly, C_12_^+^ is a notable outlier due to its significantly broader absorptions.

The geometric structure can be more directly probed by infrared (IR) spectroscopy. Whilst some vibrational frequencies have been determined from photoelectron spectra,^79^ or inferred from electronic spectra,^25,77^ fewer systems have been studied in the IR. Until recently, most studies were of smaller systems, namely C_5_^+^, C_6_, C_6_^+^ and C_8_ in solid Argon.^45,80–85^ However, it should be noted that some studies suggest IR absorptions of C_6_ and C_8_ in an Ar-matrix have been misassigned.^86^ There have been limited IR studies of monocyclic rings in the gas phase. Recently, the spectrum of cyclic C_11_ was recorded in a molecular beam,^56^ and the spectra of larger monocyclic carbon ring cations have also been reported.^18^ This study by Liu and co-workers revealed some fascinating spectral properties of C_4*n*_^+^ carbon ring cations (*n* = 5–11), whose spectra are dominated by giant infrared resonances (GIRRs) that arise due to a Kekulé-type distortion oscillating across the polyynic ring.^18^ A linear redshift of the GIRR with increasing ring size was also observed, and calculations predicted an increase in intensity for larger *n.*

The structure and spectroscopy of carbon rings are of further interest due to their relevance in astrochemistry. Given the detection of C_2_ in a carbon-rich proto-planetary nebula,^87^ C_3_ and C_5_ in carbon-rich stars,^88,89^ C_2_ and C_3_ in diffuse clouds^90,91^ and the abundance of fullerenes in certain regions of the interstellar medium,^92–98^ the question remains if intermediate carbon clusters, such as monocyclic rings, are prevalent in astrophysical environments. Whilst pure carbon clusters are not amenable to detection *via* radio astronomy due to their small dipole moment,^25^ they could instead be searched for in other spectroscopic regions, such as the visible or IR. The strong IR absorptions of C_4*n*_^+^ rings makes them enticing candidates to search for.

Although large C_4*n*_^+^ (*n* = 5–11) carbon ring cations have been recently characterised in the IR,^18^ most smaller carbon cluster cations have thus far evaded spectroscopic interrogation in the gas phase. The paucity of data limits our understanding of not only the spectral characteristics, but also the geometric structure of the smaller rings. A more complete set of spectroscopic data could also prove useful to search for such molecules in the interstellar medium.

This study presents gas phase IR spectra of C_4*n*_^+^ (*n* = 2–7) monocyclic carbon ring cations, including the first IR spectra of C_16_^+^, C_12_^+^ and C_8_^+^, by messenger spectroscopy. The experimental results are compared to DFT calculations, using a variety of methods, to aid a more thorough understanding of their geometric structure. The results are subsequently discussed in the context of astrochemistry and the potential detectability of these molecules in extra-terrestrial regions.

## 2 Methods

### 2.1 Experimental methods

Carbon cations were generated by laser ablation of a graphite rod (Goodfellow, 99.997%). The rod was rotated and translated inside a custom-made aluminium block and ablated by a focussed, frequency-doubled Nd:YAG laser (Innolas Spitlight SL600-20), operating at 20 Hz with pulse energies of ∼20 mJ/pulse. Cluster formation was promoted by admitting helium gas into the source block from a 20 Hz pulsed piezovalve. The neutral plasma generated in the source underwent a free jet expansion into a vacuum chamber, where the coldest portion of the beam was collimated by a skimmer with a 2 mm diameter orifice.

The cations were subsequently transmitted through a hexapole ion guide, after which the desired *m*/*z* was mass selected using a quadrupole mass spectrometer (Extrel GP-203D). The ions of interest were turned 90 by a quadrupole bender and loaded into a cryogenic linear quadrupole ion trap (*T*_nom_ = 4.5 K). Ions were admitted into the ion trap in the presence of dense helium buffer gas (10^15^ cm^−3^) and stored for several hundred milliseconds to cool the ions to rotational temperatures below 10 K. At these low temperatures, around 20% of the stored ions form weakly bound ion–helium complexes. These complexes readily dissociate following one photon vibrational excitation due to the low binding energy (∼100 cm^−1^) of the helium.^99^

Tunable IR radiation was generated from a 10 Hz Nd:YAG (Surelight EX) pumped optical parametric oscillator/optical parametric amplifier (OPO/OPA) system (LaserVision). Photon energies between 1500–2000 cm^−1^ were obtained by transmitting the signal and idler output of the OPO/OPA through a AgGaSe_2_ crystal. The radiation was focussed onto the ion cloud using a ZnSe lens (*f* = 25 cm) and attenuated using a ZnSe neutral density filter. Typical radiation energies were on the order of 0.1 mJ/pulse with a linewidth of 0.9 cm^−1^. The ion–helium complexes were exposed to three pulses from the OPO/OPA system on alternate trapping cycles using a mechanical shutter. The number of ions remaining with (*N*_i_) and without (*N*_0_) radiation were monitored by transmitting the ion–helium complexes through a second quadrupole mass spectrometer (Extrel GP-203D) and using a Daly-type detector.

To determine the number of background ions that did not interact with the IR radiation, the trap contents were irradiated with visible or near-IR radiation at a wavelength coincident with their electronic transitions.^25^ Ions were exposed to several pulses from an OPO (EKSPLA NT342B) with typical energies of several mJ/pulse. The number of ion–helium complexes remaining were used to determine the number of background ions (*N*_B_), attributed to contributions from other isomers or structures with the same *m/z*. The number of background ions were checked for all ions except C_8_^+^ because its electronic transitions have not yet been reported.

All spectra are plotted in terms of the relative absorption cross-section 
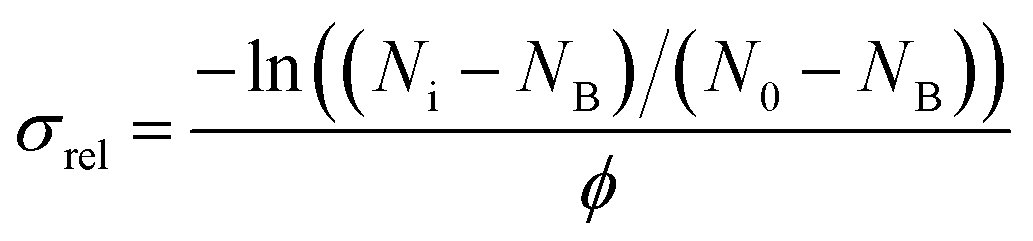
, where *N*_i_ and *N*_0_ are the number of ions extracted from the trap with and without exposure to radiation, respectively, *N*_B_ is the number of background ions and *ϕ* is the OPO/OPA fluence. The spectra have been normalised such that the strongest absorption feature presented in this study has *σ*_rel_ = 1. Due to uncertainties in the exact value of *N*_B_, the values of *σ*_rel_ are reported with an uncertainty of 20%. From the stepsize and uncertainty in the OPO/OPA calibration, band maxima are reported with an uncertainty of ± 1 cm^−1^.

### 2.2 Computational methods

All calculations presented were obtained using the Gaussian 16 software package^100^ installed at Eddie, the University of Edinburgh's research compute cluster. Starting geometries of C_4*n*_^+^ (*n* = 2–7) monocyclic rings were obtained by a force-field optimisation on Avogadro.^101^ Optimised structures and vibrational frequencies were calculated using three different functionals; B3LYP,^102–105^ PW6B95,^106^ and PBE1PBE.^107–109^ Specific details for the choice of method are discussed in detail in Section 3.2. All calculations were carried out using the def2-TZVP basis set^110,111^ with ‘tight’ convergence for the self consistent field (SCF) and geometry optimisation cycles, and a ‘superfine’ integration grid. B3LYP and PBE1PBE were also employed with Grimmes D3 dispersion correction with Becke–Johnson damping (GD3BJ).^112^ The structures were confirmed to have no imaginary frequencies or spin contamination. To further constrain the geometry of C_12_^+^, single-point energy calculations were carried out at the CCSD(T)/def2-TZVP level of theory^113^ using optimised geometries from each functional.

## 3 Results & discussion

### 3.1 Experimental observations

The photofragmentation spectra of carbon clusters C_4*n*_^+^–He (*n* = 2–7, herein denoted C_4*n*_^+^) are presented in Fig. 2, and the spectral characteristics are detailed in Table 1. Two trends are immediately apparent from Fig. 2 – there is a decrease in IR intensity, and a blueshift of the IR-active mode, as the number of carbon atoms in the ring decreases. There are also clear differences in the spectra of C_24_^+^, where multiple absorptions are observed, and in C_8_^+^, which does not follow the aforementioned linear trends and displays a much broader, asymmetric absorption profile. From these observations, it would appear that C_28_^+^ to C_16_^+^ share a common geometric structure, which deviates slightly for C_12_^+^ and changes substantially for C_8_^+^.

**Fig. 2:**
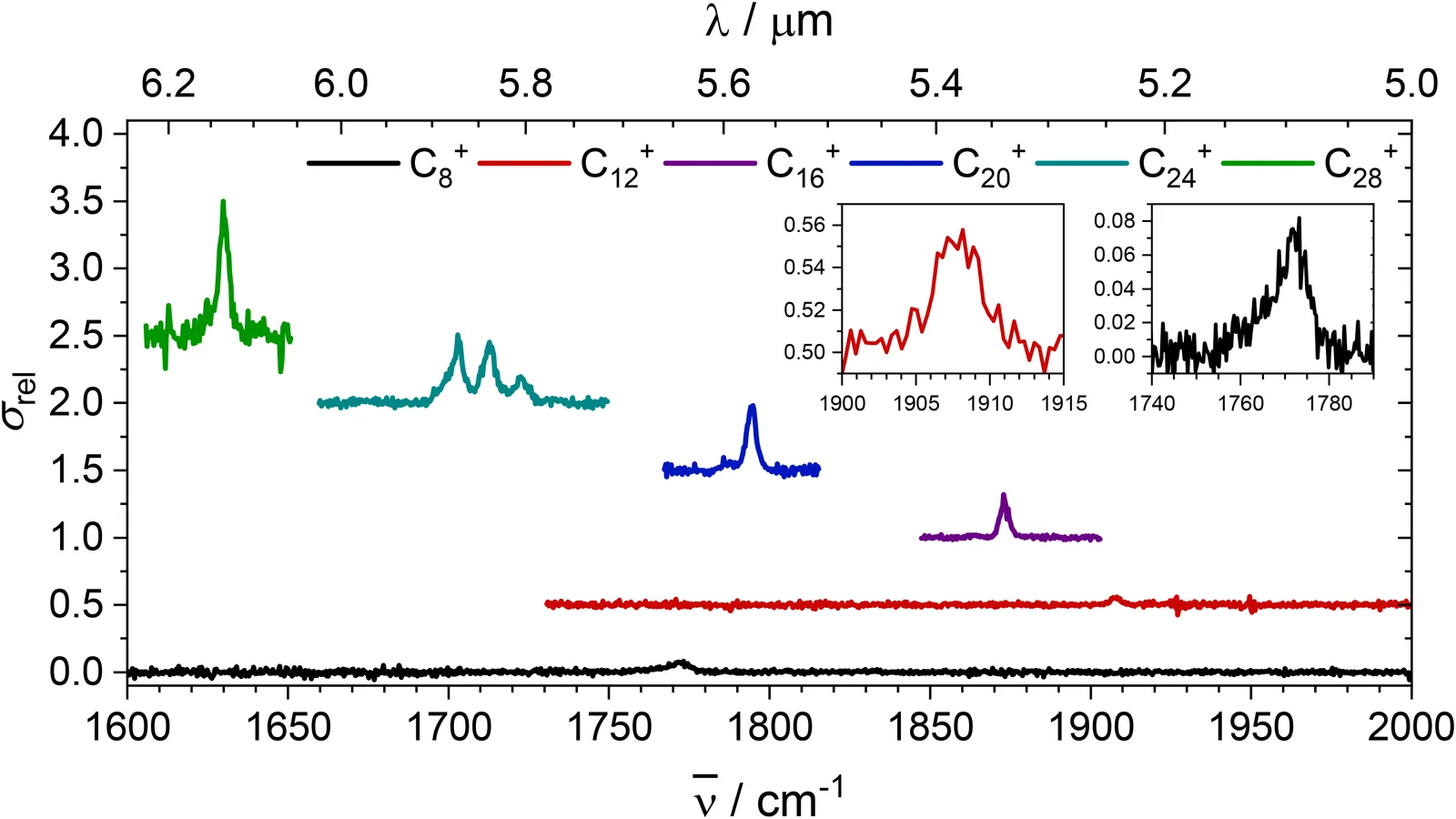
Photofragmentation spectra of C_4*n*_^+^–He (*n* = 2–7). The inset panels show the strongest vibrational transitions of C_12_^+^–He (red, left) and C_8_^+^–He (black, right). The spectra are offset vertically, in multiples of 0.5, for clarity. C_8_^+^–He shows a distinctly different spectral profile from other C_4*n*_^+^–He clusters (see text).

**Table 1:** A comparison of the experimental characteristics of C_4*n*_^+^ (*n* = 2–7) recorded using He-tagging (this work) and N_2_-tagging.^18^ Weak vibrational transitions are written in italics

Molecule	N_2_-tag	This work (He-tag)		
	Band maximum/cm^−1^	Band maximum/cm^−1^	FWHM/cm^−1^	*σ*_rel_
C_8_^+^	—	1773	9	0.08
C_12_^+^	—	1908	3	0.06
C_16_^+^	—	*1864*	2	0.02
		1873	3	0.32
C_20_^+^	—	*1788*	3	0.06
	1801	1795	3	0.48
C_24_^+^	1674	*1672*	7	0.03
	1705	1703	5	0.51
	1715	1713	4	0.45
	1724	1723	4	0.20
C_28_^+^	1634	1630	3	1.00

The distinct increase in IR intensity with increasing *n* has been attributed to two factors. Firstly, as the ring size increases, the physical displacement of oscillating charge becomes larger, meaning the total change in dipole moment is increased. Secondly, as the ring size is made bigger, the energy gap between the frontier orbitals decreases, intensifying any coupling between electronic and vibrational motions.^18^ This rapid increase of IR intensity was predicted by calculations in ref. 18, and is experimentally verified in this study.

As seen in Table 1, the band maxima of C_20_^+^, C_24_^+^ and C_28_^+^ are in close agreement between He-tagging (this work) and N_2_-tagging spectroscopy.^18^ The band maxima are typically red-shifted by ∼3 cm^−1^ in the He-tagging spectra compared to the N_2_-tagging spectra, which is attributed to the stronger binding, and larger perturbative effect, of the N_2_-tag. The full width at half maxima (FWHM) of the spectra in this work are generally smaller than the N_2_-tagged spectra (∼6 cm^−1^), which is likely due to the narrower linewidth of the OPO used (0.9 cm^−1^ compared to 6 cm^−1^ in ref. 18). Due to the mass resolution used, the spectra presented may have a small contribution from ions containing a ^13^C isotope which could contribute to the observed ∼3 cm^−1^ widths. To estimate this broadening effect, the change in vibrational frequency between ^12^C_28_^+^ and ^13^C^12^C_27_^+^ was calculated. The average first order shift of the totally symmetric vibrations of C_28_^+^ can be approximated as 
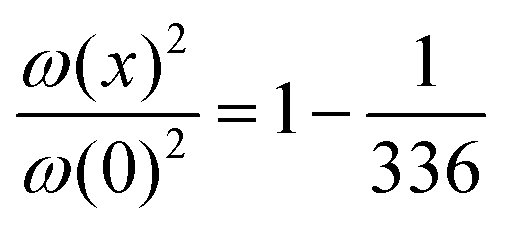
, where *ω*(*x*) is the vibrational frequency of the ion containing a single ^13^C isotope.^114^ Although the Kekulé-type vibrational mode is not totally-symmetric, using the above formula yields a band maximum of ^13^C^12^C_27_^+^ at approximately 1628 cm^−1^ compared to 1630 cm^−1^ for ^12^C_28_^+^. Whilst this is not a negligible shift, there is no clear change in the FWHM as the size of the ring changes to suggest the widths are dominated by isotope effects. Furthermore, given that many helium-tagged spectra of polycyclic aromatic hydrocarbon (PAH) and carbon cluster species have been observed to have a similar FWHM of around 3 cm^−1^,^115–118^ the broadening observed is expected to be dominated by the short picosecond lifetime of the ion–helium complex. An exception to this is the IR feature of C_8_^+^ which has a FWHM of 9 cm^−1^ and a shoulder appearing to the red of the band maximum, meaning contributions from ^13^C^12^C_7_^+^ cannot be ruled out. These observations are discussed in more detail in Section 3.2. It should be noted that whilst there will be a small perturbation of the band maxima due to the presence of the helium-tag, this is expected to be small compared to the widths of the features. Previous studies from our laboratory on a number of PAH and related systems have estimated this perturbation to be approximately one wavenumber.^115–118^

The relative cross-section of the C_8_^+^ transition may be underestimated by assuming that 100% of the ions probed were the monocyclic ring isomer. The percentage of background ions is not known for C_8_^+^ because gas phase spectroscopic data is not available to enable saturation of its electronic transition. Although no other strong IR features were observed between 1500–2000 cm^−1^, given the small size of this cluster, it is plausible that other isomers, such as linear chains, could be generated from the laser ablation source. The percentage of monocyclic carbon rings obtained from background ion measurements agree qualitatively with previous ion mobility studies.^11^

The spectrum of C_24_^+^ shows multiple strong peaks separated by 10 cm^−1^. Due to the very high intensity of the GIRRs, it is believed these additional features are caused by Fermi-resonances.^18,119^ Whilst the same four IR features are also observed in the N_2_-tagged spectrum,^18^ it is worth noting that the relative intensities of these differ significantly between the two studies. In this work, the lower-energy feature at 1703 cm^−1^ is the most intense mode. This is not believed to be due to saturation effects because the band at 1672 cm^−1^ is significantly weaker than in the N_2_-tagged spectrum. It is plausible that the smaller perturbation of the helium tag, relative to the nitrogen tag, could cause a measurable change in the amount of intensity stealing from these Fermi resonances. Further inspection of C_20_^+^ and C_16_^+^ also show the presence of weak shoulder features, which are listed in italics in Table 1. These are also attributed to Fermi resonances. It is worth noting that the cross-section of the strongest C_24_^+^ transition is similar to C_20_^+^, and significantly weaker than C_28_^+^, providing additional evidence of intensity stealing from the strongest vibrational mode.

In order to completely rule out the presence of other C_24_^+^ isomers causing the additional vibrational features, the ion cloud was continuously irradiated at 1100 nm by an EKSPLA NT342B OPO with pulse energies of several mJ/pulse. This was used to excite the electronic transition of monocyclic C_24_^+^–He, removing these ion–helium complexes from the ion trap.^25^ The IR OPO/OPA system was then scanned over this wavelength range, and no IR features were observed. This confirms that all the observed transitions are from the same monocyclic carbon ring isomer, as expected.

A key observation is the clear linear blueshift of the IR mode with decreasing ring size from C_28_^+^ to C_16_^+^, with an average value of approximately 80 cm^−1^ between successive 4*n* carbon clusters. Interestingly, this trend breaks down at C_12_^+^, which is only blueshifted 35 cm^−1^ from C_16_^+^. Notably, C_8_^+^, with a band maxima at 1774 cm^−1^, does not follow this linear trend at all. To aid explanation of these observations, a variety of geometry and frequency analyses were performed, which are discussed and compared to the experimental data below.

### 3.2 Comparison to theory

The choice of DFT functionals used in this study proceeds from information provided by a perspective of best-practice by Bursch and colleagues.^120^ Hybrid functionals PW6B95 and PBE1PBE, with the def2-TZVP basis set, were chosen to balance computational cost and accuracy. Calculations were also done using the B3LYP functional, which is discussed to have a proven track record for the computation of IR spectra.^120^ The choice of the B3LYP functional also facilitates comparisons with the work by Liu and co-workers.^18^

In Fig. 3a, the experimental band maxima are compared to the strongest IR mode predicted from harmonic vibrational frequency calculations using these three functionals. All give close agreement with one another for C_20_^+^ and larger 4*n* clusters, however, the energy of the vibrational mode is systematically overestimated. This is likely because no anharmonicity has been taken into account. Inspection of the bond length alternation (BLA) and bond angle alternation (BAA), plotted in Fig. 3b and c respectively, indicate similar structures are obtained. Here, a BLA of approximately 0.08–0.09 Å that decreases slowly as the ring size increases is predicted, with a BAA of 0°. These structures correspond to point groups of 
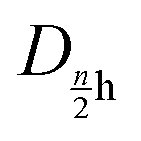
 symmetry, where *n* is the number of carbon atoms in the monocyclic ring. These structures are slightly higher symmetry than those calculated by Liu and co-workers^18^ using B3LYP/cc-pVDZ, who obtain a 
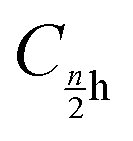
 symmetry arrangements with a similar BLA but a small BAA.

**Fig. 3:**
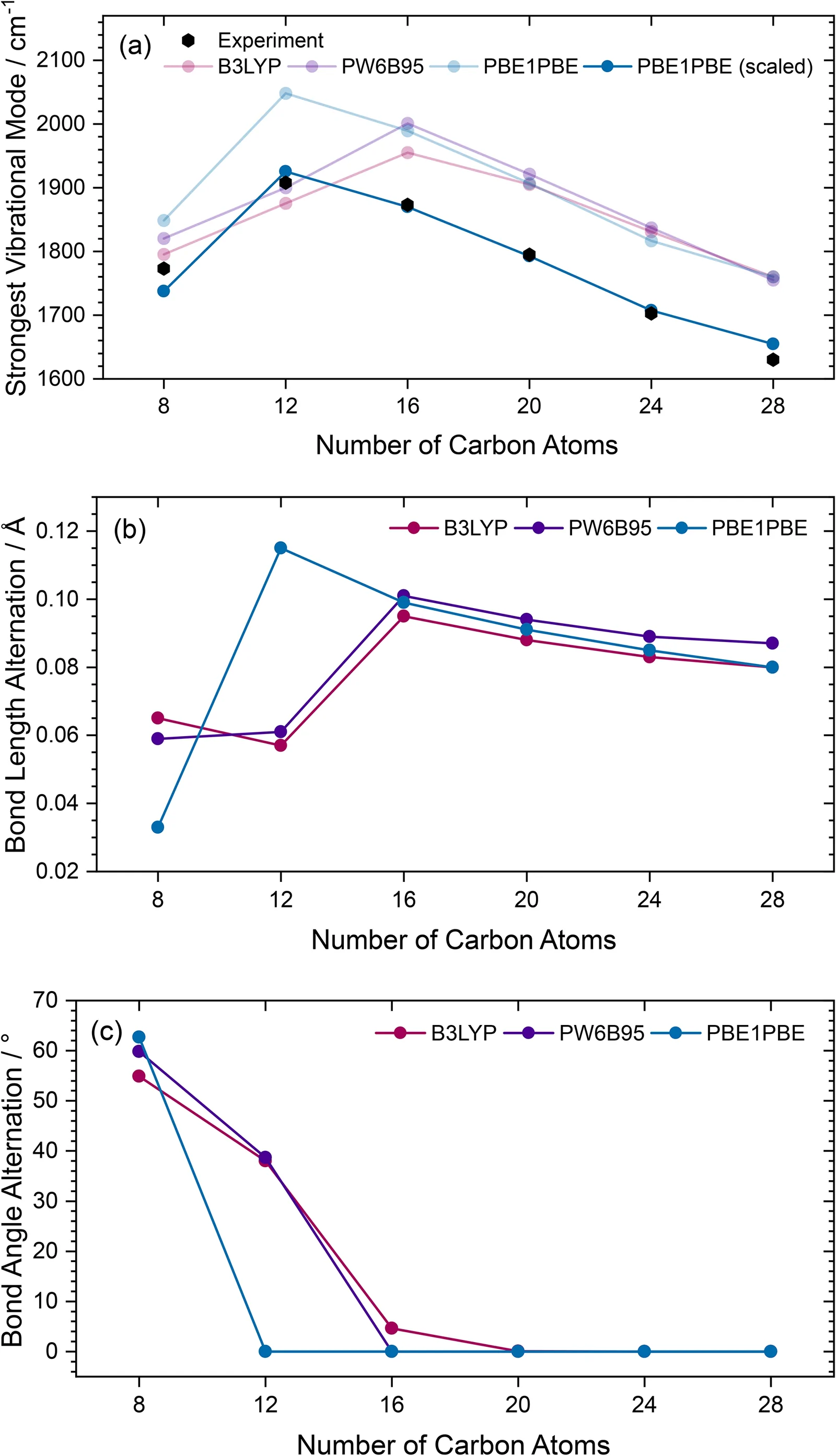
Comparison between the experimental characteristics and theoretical structure of C_4*n*_^+^ monocyclic rings using different functionals with the def2-TZVP basis set. Note the BLA and BAA of C_8_^+^ calculated using the PBE1PBE functional is the average value, since this structure exhibits lower (*C*_2v_) symmetry. (a) Comparison of the experimental band maxima to the most intense IR transition predicted by theory. Overall, PBE1PBE best follows the experimentally-observed trends and is additionally plotted with a 0.94 scaling factor. (b) Plot of the bond length alternation (BLA) predicted using different functionals for a series of 4*n* carbon clusters. Comparison to Fig. 3a shows similar trends between BLA and the frequency of the most intense IR mode. (c) Plot of the bond angle alternation (BAA) predicted using different functionals for a series of 4*n* carbon clusters. Calculations predict larger clusters to have 
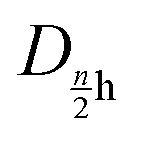
 symmetry, however, results for smaller clusters show discrepancies.

Larger discrepancies between the chosen functionals arise for smaller 4*n* clusters. For C_16_^+^, B3LYP deviates slightly from the other two functionals, predicting a BAA of 4.6°, and therefore a structure with *C*_8h_ symmetry. The calculated frequency of the GIRR is no longer linearly-blueshifted, which is not in agreement with experiment.

More significant differences become apparent for C_12_^+^. PBE1PBE predicts a higher energy vibrational mode with a *D*_6h_ structure, containing a large BLA and a BAA of 0° (see structure C in Fig. 1). Meanwhile, B3LYP and PW6B95 give *C*_6h_ structures with a smaller BLA and a significant BAA (structure D in Fig. 1). B3LYP and PW6B95 fail to model the overall trends, as both these methods predict a redshift in the vibrational mode from C_16_^+^ to C_12_^+^. Only PBE1PBE correctly predicts the experimentally-observed blueshift. Furthermore, the calculated IR intensities from this functional are more representative of the experimental spectra. This can be more clearly seen in Fig. S1–S3 in the supporting information (SI). This suggests the structure of C_12_^+^ observed in this experiment has similarities to the geometry obtained from the PBE1PBE calculation, which is overall similar in structure to larger C_4*n*_^+^ carbon rings. Comparison of Fig. 3a and b suggests some relationship between the frequency of the strongest vibrational mode and the BLA, which could be explored further in a dedicated computational study.

To further constrain the geometry of C_12_^+^, single-point energy calculations were carried out at the CCSD(T)/def2-TZVP level of theory using the optimised geometries for each functional. The D_6h_ structure, obtained using the PBE1PBE functional, was found to be 17.6 and 14.8 kJ mol^−1^ lower in energy than the *C*_6h_ structures calculated using the PW6B95 and B3LYP functionals, respectively. This energy gap gives some additional evidence that the *D*_6h_ structure is more representitive of the true ground state geometry. It should be noted that whilst the CCSD(T) calculation using the PBE1PBE optimised geometry has a T1 diagnostic of 0.018, indicating minor multireference effects,^103^ calculations on the other two structures had larger T1 diagnostic values of 0.037 and 0.038 for PW6B95 and B3LYP, respectively. This highlights the need for multireference calculations, beyond DFT, to adequately describe open shell, monocyclic carbon ring cations. Wavefunction stability checks were also performed for each calculation using the ‘stable’ keyword on Gaussian 16. This revealed a slight internal instability for the *D*_6h_ geometry of −2.7 kJ mol^−1^. However, relaxing the wavefunction and re-optimising the geometry led to severe spin contamination. Given the small magnitude of the energy instability, this spin-contaminated state could be an artefact of (unrestricted) DFT methods, which reinforces the need for multireference calculations to adequately describe C_12_^+^.

Previous investigations have revealed the electronic spectrum of C_12_^+^ to be broadened relative to other C_4*n*_^+^ clusters, which cannot be solely explained by the rotational envelope of doublet–doublet transitions of monocyclic rings of this size.^25,77^ Assuming the large width arises due to lifetime broadening, the excited state lifetime of C_12_^+^ must be notably shorter than other carbon ring cations. Future calculations could also investigate the electronic structures of the *C*_6h_ and *D*_6h_ geometries and probe if one of these structures better explains the observed lifetime broadening, for example due to close-lying electronic states that facilitate fast, non-radiative relaxation.

The optimised geometries of C_8_^+^ all show a large change in geometric structure from the larger rings, with a pronounced BAA and a reduced BLA. However, there are some discrepancies in the optimised geometries. Whilst B3LYP and PW6B95 predict a *C*_4h_ structure, similar to previous theoretical studies,^17,121^ PBE1PBE has an optimised geometry in *C*_2h_ symmetry. Therefore, the given BLA and BAA values for this functional are instead average values. The *C*_2h_ structure shows a loss of degeneracy, with two vibrational features separated by approximately 80 cm^−1^ predicted. The stronger of the two modes is plotted in Fig. 3a.

Interestingly, only one asymmetric feature was observed in the experimental spectrum. The rotational profile of the C_8_^+^ absorption was simulated on PGOPHER using calculated rotational constants from the *C*_2h_ (PBE1PBE) and the *C*_4h_ (PW6B95) structures at a rotational temperature of 10 K.^47^ Surprisingly, neither of these simulations managed to reproduce the observed asymmetry of this absorption feature. This was also true even after allowing the excited state rotational constants to vary by a few percent. The best way to explain the observed asymmetry is by simulating two vibrational features on PGOPHER using the PBE1PBE calculated rotational constants. The origin and intensity of both features were allowed to vary, along with the Lorentzian convolution, which gave two features separated by 8 cm^−1^. This simulation can be seen in Fig. 4a. It is, therefore, suggested that the profile of C_8_^+^ is explained by both the rotational envelope and a loss in degeneracy of the vibrational mode as the molecule distorts slightly from *C*_4h_ to *C*_2h_ symmetry. This loss in degeneracy appears to be significantly overestimated using the PBE1PBE functional, and not captured by the other two functionals. A tag-free technique, such as two colour fragmentation, may better resolve individual modes by reducing the contribution of lifetime broadening from the ion–helium complex. As discussed in Section 3.1, it is possible that isomeric broadening may also contribute to the asymmetric profile of this absorption feature.

**Fig. 4:**
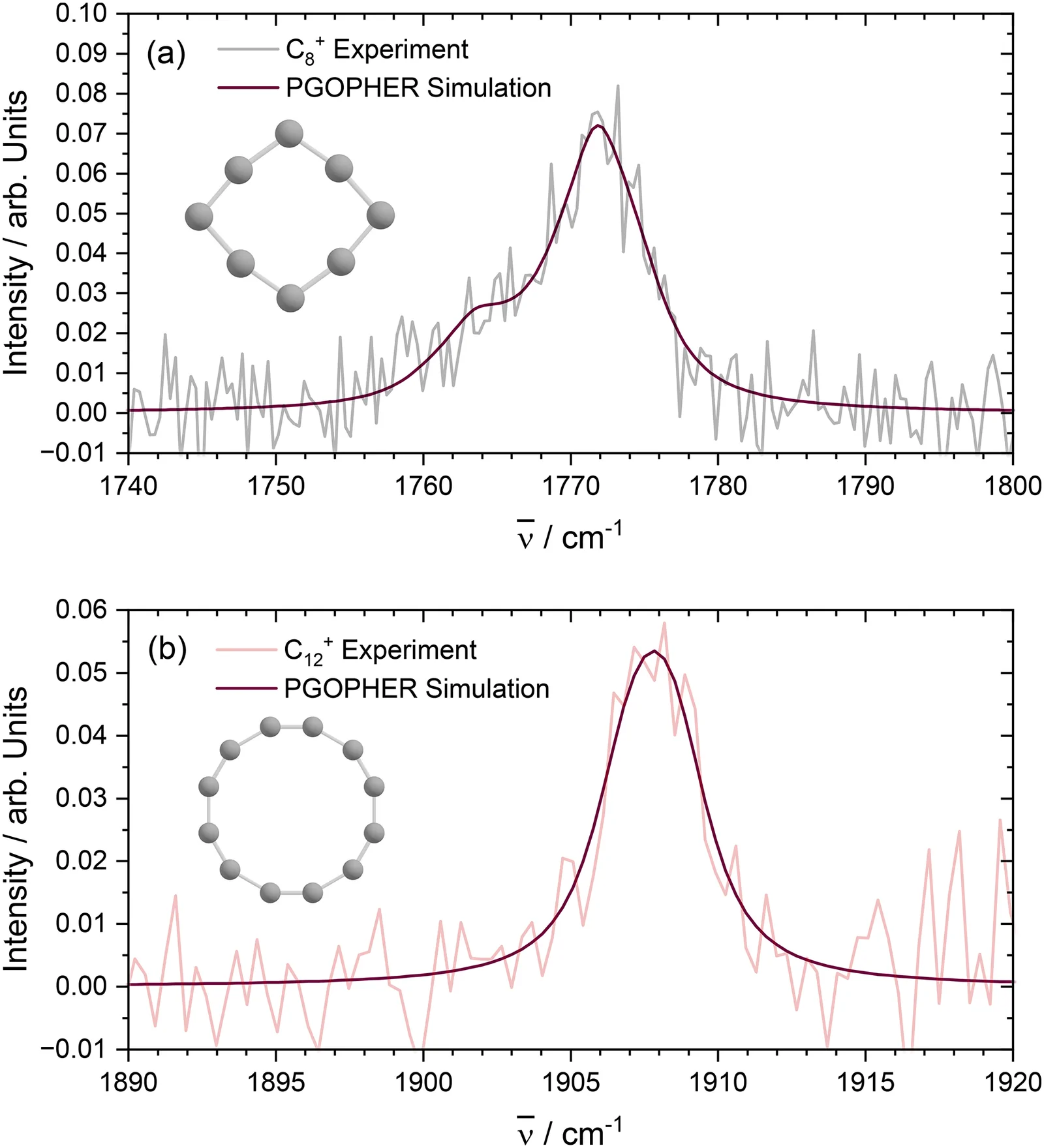
Simulation of rotational envelopes of C_8_^+^ and C_12_^+^ at 10 K using PGOPHER. The images are optimised geometries calculated using the PBE1PBE/def2-TZVP functional and basis set combination. (a) Simulation of the rotational envelope of C_8_^+^ using calculated rotational constants from PBE1PBE/def2-TZVP. The best fit to the experiment is realised when two vibrational transitions, separated by 8 cm^−1^, are simulated. (b) Simulation of the rotational envelope of C_12_^+^ using calculated rotational constants from PBE1PBE/def2-TZVP.

For completeness, the rotational envelope was also simulated using the PBE1PBE calculated rotational constants for C_12_^+^, which is in good agreement with the experiment, as seen in Fig. 4b. The change in rotational constants from the *D*_6h_ to *C*_6h_ structures is small, meaning it is not possible to resolve these structures from the experiment by simulating the rotational profiles. This is especially true given the lifetime broadening of the vibrational features.

The different predictions of PBE1PBE compared to the other functionals tested could be due to the non-empirical nature of this functional. A dedicated theoretical study could be completed to explore this functional-dependent nature further. Overall, by scaling the calculated frequencies from the PBE1PBE functional by the average difference from the experimentally-obtained values (0.94), a close match to the experiment is obtained, as shown in the dark blue trace in Fig. 3a. The attachment of the helium tag is expected to cause minimal perturbation to the structure and vibrational frequencies. Calculations on C_8_^+^–He, using the same three functionals, suggest the helium has a preferential site directly above the carbon ring. All the calculations predict a negligible (<0.5 cm^−1^) redshift of the strongest vibrational mode.

One thing is clear from this study: there is a definitive change in structure from a higher-symmetry, polyynic structure, to a lower-symmetry, alternating bond angle configuration, as the C_4*n*_^+^ ring size decreases. From this work, it appears this change happens around C_12_^+^, but it is particularly challenging to model this with DFT methods. The large discrepancies between the calculated structures of C_12_^+^ make this ion particularly enticing for a future theoretical study.

### 3.3 Astrochemical implications

Monocyclic carbon ring cations containing 4*n* carbon atoms exhibit very strong transitions in the IR, which have the potential to be detected in the interstellar medium (ISM). A plausible region where carbon ring cations may be present is in carbon-rich and hydrogen-poor objects. Possible candidates, therefore, include planetary nebulae, such as SMP SMC 16, SMP LMC 56 or Tc 1. For cations to be present, there must be enough UV radiation to ionise molecules. Regions of planetary nebulae closer to the central star may be promising to search for these ions.^122^ However, harder radiation fields are often accompanied by higher electron densities, and it is the balance between these that determines the charge distribution in planetary nebulae, complicating where to search for these molecules.^116^ Another promising candidate object to search for monocyclic carbon ring cations is NGC 7023. This reflection nebula has a strong radiation field and its photodissociation regions have been well-studied.^123^ Pure carbonaceous molecules, such as fullerenes, have already been detected in many of these astrophysical objects,^92,93,97,122^ and it is possible that similar pure carbon rings may also be in these regions. Detection of these smaller carbonaceous structures may provide new information on the formation or destruction pathways of fullerenes.^124^

A further complication is many IR-active species are detected in space from emission, rather than absorption. To the best of our knowledge, although many studies have been published for polycyclic aromatic hydrocarbon molecules,^125^ dedicated studies on emission have not been carried out on carbon rings. Whilst these are strongly desired, it should be noted that some carbon cluster ions have been studied in ion storage ring facilities.^126–130^ These investigations have demonstrated the importance of recurrent fluorescence in stabilising these ions where, below a certain internal energy threshold, IR emission is likely to be an important cooling mechanism. Interestingly, the cooling timescales can vary by orders of magnitude between some of these ions, presumably due to the presence (or lack of) low-lying electronic states.^128^ Some larger carbon cluster cations show impressive stabilising effects, even above 13.6 eV, which may increase their survival probability in the ISM such as in HI regions.^130^

Given the lack of dedicated studies on IR emission for carbon ring cations, in general, it can be assumed that IR emission causes a pronounced low-frequency wing due to cascade emission from higher vibrational states. However, many factors can influence the emission profile, such as the energy of the initially absorbed UV photon or the rotational temperature.^125^ On the other hand, given the strength of the IR features, some of which have comparable intensities to electronic transitions, detection by absorption should not be completely ruled out.

A complication in searching for monocyclic carbon ring cations in space is they only have one absorption feature, meaning a match between laboratory data and astronomical observation cannot be ruled out as just a coincidence. However, it is plausible that a distribution of ring sizes may be present in the same region of the ISM. Assuming there is an equal contribution from all the carbon clusters seen in Fig. 2, this would give the absorption spectrum presented in the black trace in Fig. 5 In reality, these ions are not going to be present in identical proportions, so the relative intensities would likely differ significantly based on relative abundances.

**Fig. 5:**
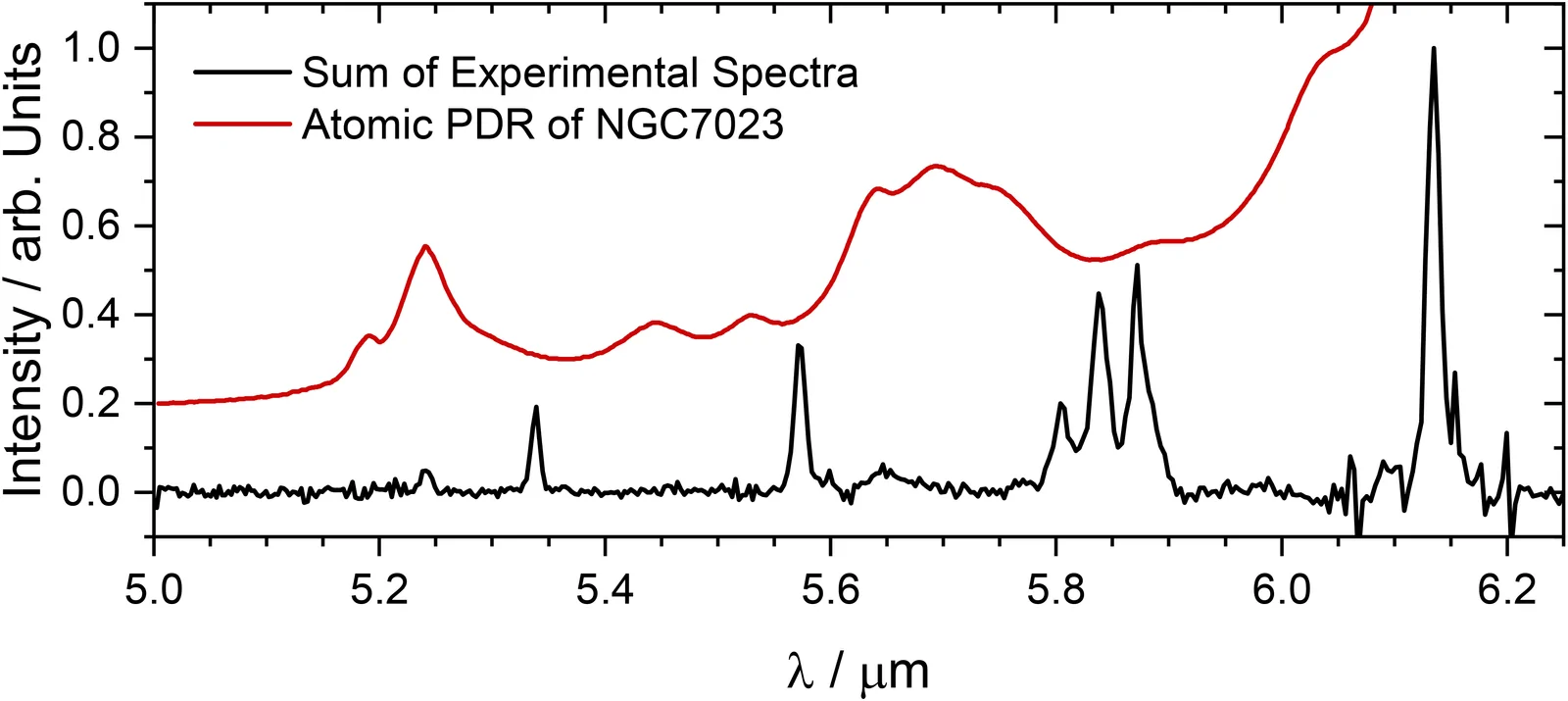
Comparison of emission spectra from the atomic photodissociation region (PDR) of the reflection nebula NGC 7023 (red, digitised from ref. 123) to laboratory spectra (black). The experimental spectrum consists of an equally-weighted sum of C_4*n*_^+^ (*n* = 2–7) carbon clusters with the most intense peak normalised to 1.

For comparison, the emission spectra from the atomic photodissociation region of NGC 7023, digitised from ref. 123, is presented in the red trace in Fig. 5 The comparison shows no discernable features which can be attributed to these carbon ring cations based on this particular spectrum. It is worth noting that no C_60_ was observed in this particular study, and JWST observations closer to the star, where C_60_ has been observed previously with the Spitzer space telescope, may be a more promising region to search for pure carbonaceous clusters in NGC 7023.

## 4 Conclusions

In this study, gas phase IR absorption spectra of a series of monocyclic carbon ring cations (C_4*n*_^+^, *n* = 2–7), including the first IR spectra of C_16_^+^, C_12_^+^ and C_8_^+^, have been presented. From these data, two trends are immediately apparent. Firstly, as the number of carbon atoms decreases, the IR intensity reduces. This is believed to arise from a smaller physical displacement of oscillating charge, and a reduction in coupling between electronic and vibrational motion as the energy gap between the frontier orbitals increases. The relative intensities of these IR features have been experimentally-determined for the first time. Secondly, as the number of carbon atoms decreases, the strongest vibrational mode linearly blueshifts from C_28_^+^ to C_16_^+^. The blueshift is smaller in magnitude from C_16_^+^ to C_12_^+^, and C_8_^+^ shows no relation to any of the aforementioned trends.

DFT calculations suggest larger rings, namely C_16_^+^ and above, adopt polyynic structures, with equal bond angles around the ring. Calculations using various functionals show large discrepancies for the geometry of C_12_^+^. However, comparison between the experimental, gas phase data with calculated IR intensities and energies suggests that C_12_^+^ likely adopts a similar structure to the larger rings with a large BLA. Further multireference calculations will be required to confirm the exact geometric structure of this ion.

A comparison between the experiment and calculations suggests that C_8_^+^ adopts a less symmetrical arrangement, with a large BAA and a smaller BLA. This large structural change is expected to explain why this particular ion no longer follow the trends observed for the larger clusters. Simulation of the rotational envelope also suggest this structure is slightly distorted from *C*_4h_ to *C*_2h_ symmetry. The extent of this distortion shows some disagreement amongst the calculated structures. A tag-free technique, such as two colour fragmentation, may better reveal this underlying structure, since the current helium-tagged spectra are hindered by lifetime broadening effects.

The intense IR features observed make these ions promising candidates to search for in the interstellar medium. However, confirmation of their presence is frustrated since each species only has one IR active mode, preventing any definite assignment. Astronomical searches should, therefore, search for spectroscopic signatures from multiple carbon rings in the same astrophysical environment to help confirm any detection.

## Author contributions

T. E. Douglas-Walker: investigation (lead); formal analysis (lead); visualisation (lead); writing – original draft (lead); writing – review & editing (equal). A. Debnath: investigation (supporting); formal analysis (supporting); writing – review & editing (equal). O. O’Neill: investigation (supporting); writing – review & editing (equal). E. K. Campbell: supervision (lead); funding acquisition (lead); resources (lead); writing – review and editing (equal).

## Conflicts of interest

There are no conflicts to declare.

## Supplementary Material

CP-028-D6CP03280J-s001

## Data Availability

The data supporting this article are included within the submitted manuscript. Raw data used to plot the figures is available from the corresponding author upon reasonable request. Supplementary information (SI) is available. Supplementary information: harmonic frequency calculations of C_4*n*_^+^ monocyclic rings (*n* = 2–7) using the PBE1PBE, B3LYP and PW6B95 functionals with the def2-TZVP basis set. See DOI: https://doi.org/10.1039/d6cp03280j.
