# Supplementary material for: Unravelling the structure of monocyclic carbon ring cations using vibrational spectroscopy
Source: Phys Chem Chem Phys. 2026 Oct 5;28(40):24796–807. doi: 10.1039/d6cp03280j (PMC13637026; doi:10.1039/d6cp03280j)
Supplement: Supplementary file 1 [file CP-028-D6CP03280J-s001.pdf]

Supplementary Information (SI) for Physical Chemistry Chemical Physics.
This journal is © the Owner Societies 2026
Supplementary Information (SI) for Physical Chemistry Chemical Physics.
This journal is © the Owner Societies

Supplementary Information: Unravelling the Structure of Monocyclic Carbon Ring Cations using Vibrational Spectroscopy

Thomas E. Douglas-Walker, Amit Debnath, Olivia O'Neill, and Ewen K. Campbell*

School of Chemistry, The University of Edinburgh, Joseph Black Building, David Brewster Road, King's Buildings, Edinburgh, EH9 3FJ, Scotland, United Kingdom

E-mail: e.k.campbell@ed.ac.uk

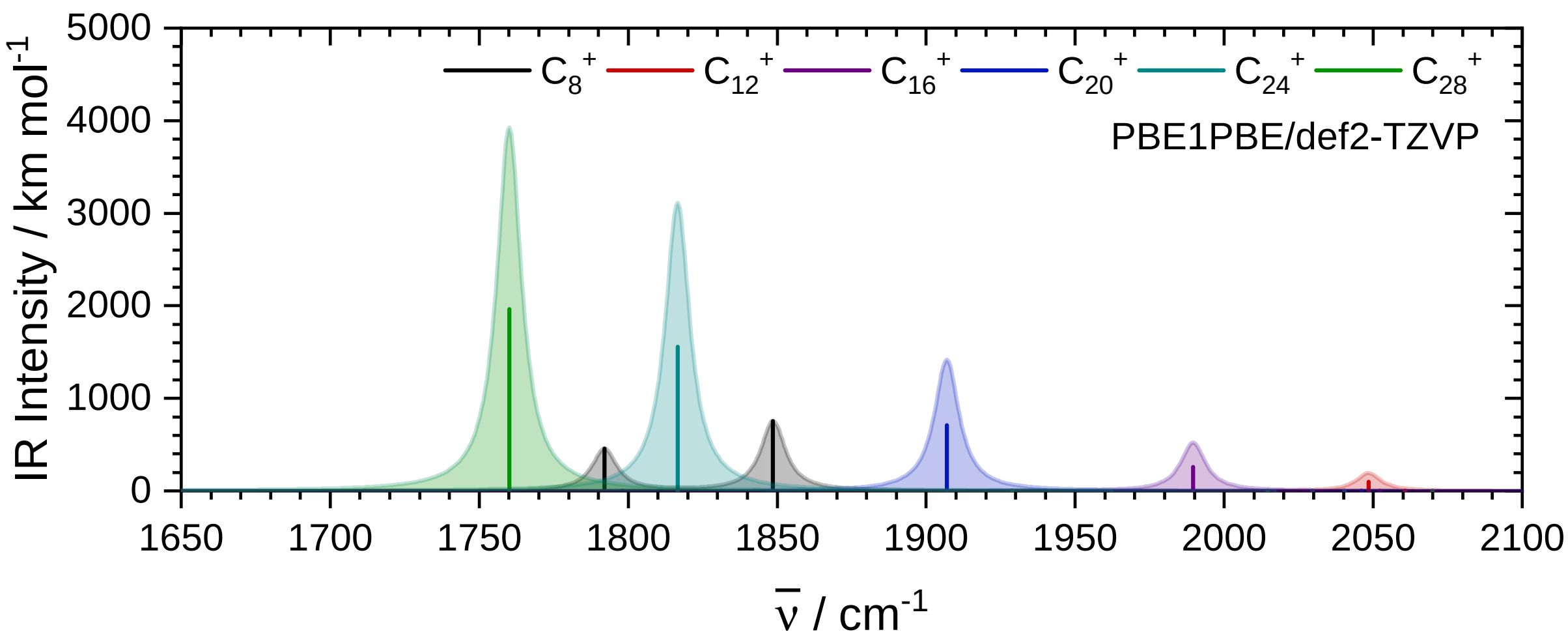

Figure S1: Calculated IR transitions of C_{4n}^+ monocyclic rings ($n = 2 - 7$) using the PBE1PBE/def2-TZVP functional and basis set. Stick spectra are convolved with Lorentzian functions with a FWHM of 10 cm^{-1}.

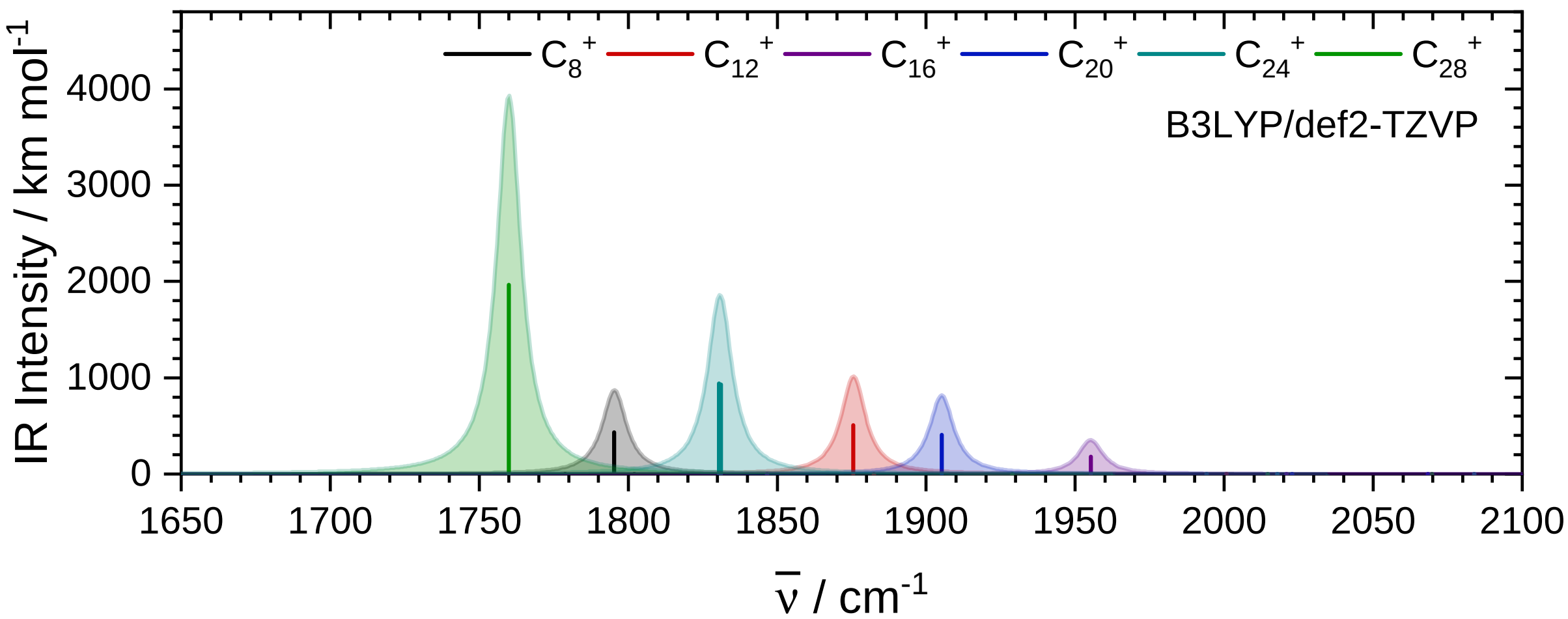

Figure S2: Calculated IR transitions of C^{+}_{4n} monocyclic rings ($n = 2 - 7$) using the B3LYP/def2-TZVP functional and basis set. Stick spectra are convolved with Lorentzian functions with a FWHM of $10\,\mathrm{cm}^{-1}$.

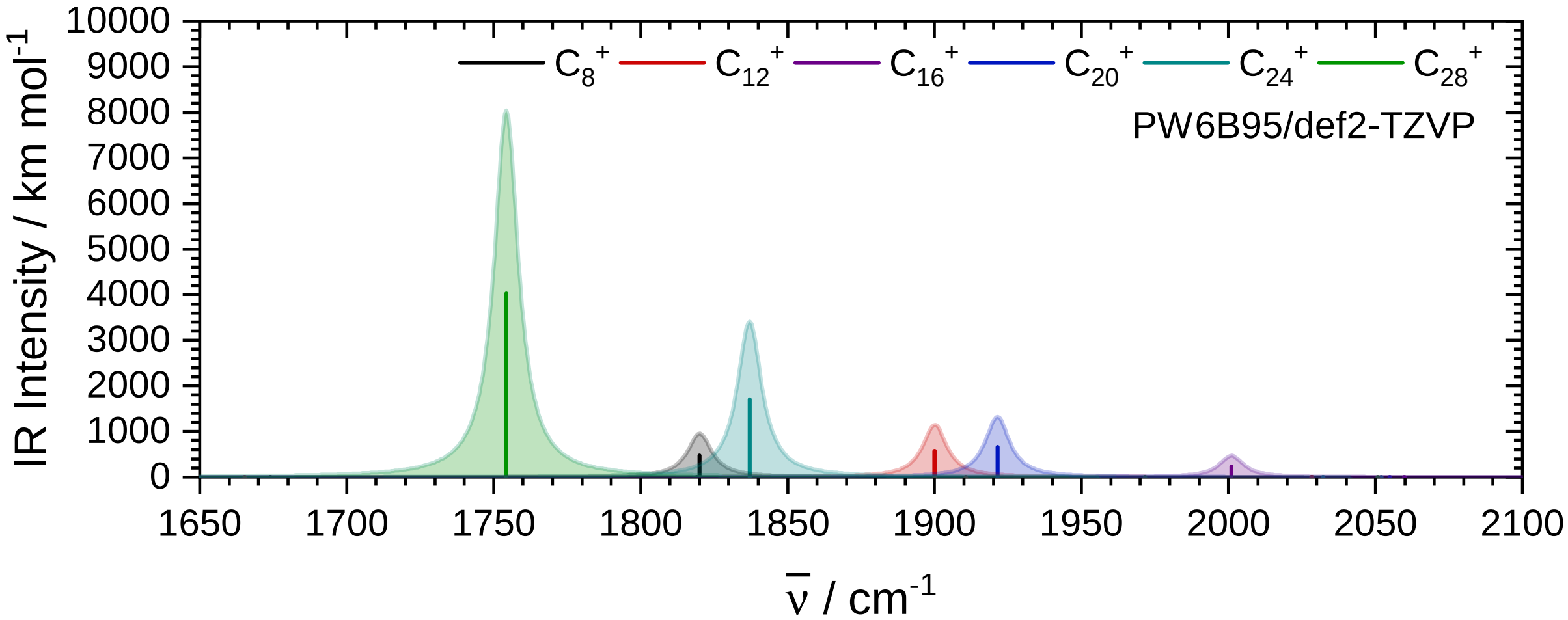

Figure S3: Calculated IR transitions of C^{+}_{4n} monocyclic rings ($n = 2 - 7$) using the PW6B95/def2-TZVP functional and basis set. Stick spectra are convolved with Lorentzian functions with a FWHM of $10\,\mathrm{cm}^{-1}$.
